# Preparation of high-resolution micro/nano dot array by electrohydrodynamic jet printing with enhanced uniformity

**DOI:** 10.1038/s41598-024-57225-5

**Published:** 2024-03-23

**Authors:** Yiwei Jin, Ziwei Zhao, Jiankui Chen, Wei Chen, Guozhen Wang, Zhouping Yin

**Affiliations:** The State Key Laboratory of Intelligent Manufacturing Equipment and Technology, School of Mechanical Science and Engineering, HuaZhong University of Science and Technology, Wuhan, 430070 China

**Keywords:** Engineering, Nanoscience and technology

## Abstract

The high-resolution array is the basic structure of most kinds of microelectronics. Electrohydrodynamic jet (E-Jet) printing technology is widely applied in manufacturing array structures with high resolution, high material compatibility and multi-modal printing. It is still challenging to acquire high uniformity of printed array with micro-nanometer resolution, which greatly influences the performance and lifetime of the microelectronics. In this paper, to improve the uniformity of the printed array, the influence of each parameter on the uniformity of the E-jet printed dot array is studied on the cobuilt NEJ-E/P200 experimental platform, finding the applied voltage plays the most important role in maintaining the uniformity of the printed array. By appropriately adjusting the printing parameters, the dot arrays with different resolutions from 500 pixels per inch (PPI) to 17,000 PPI are successfully printed. For arrays below and over 10,000 PPI, the deviations of the uniformity are within 5% and 10% respectively. In this work, the dot array over 15,000 PPI is first implemented using E-jet printing. The conclusions acquired by experimental analysis of dot array printing process are of great importance in high resolution array printing as it provides practical guidance for parameters adjustment.

## Introduction

**Figure 1:**
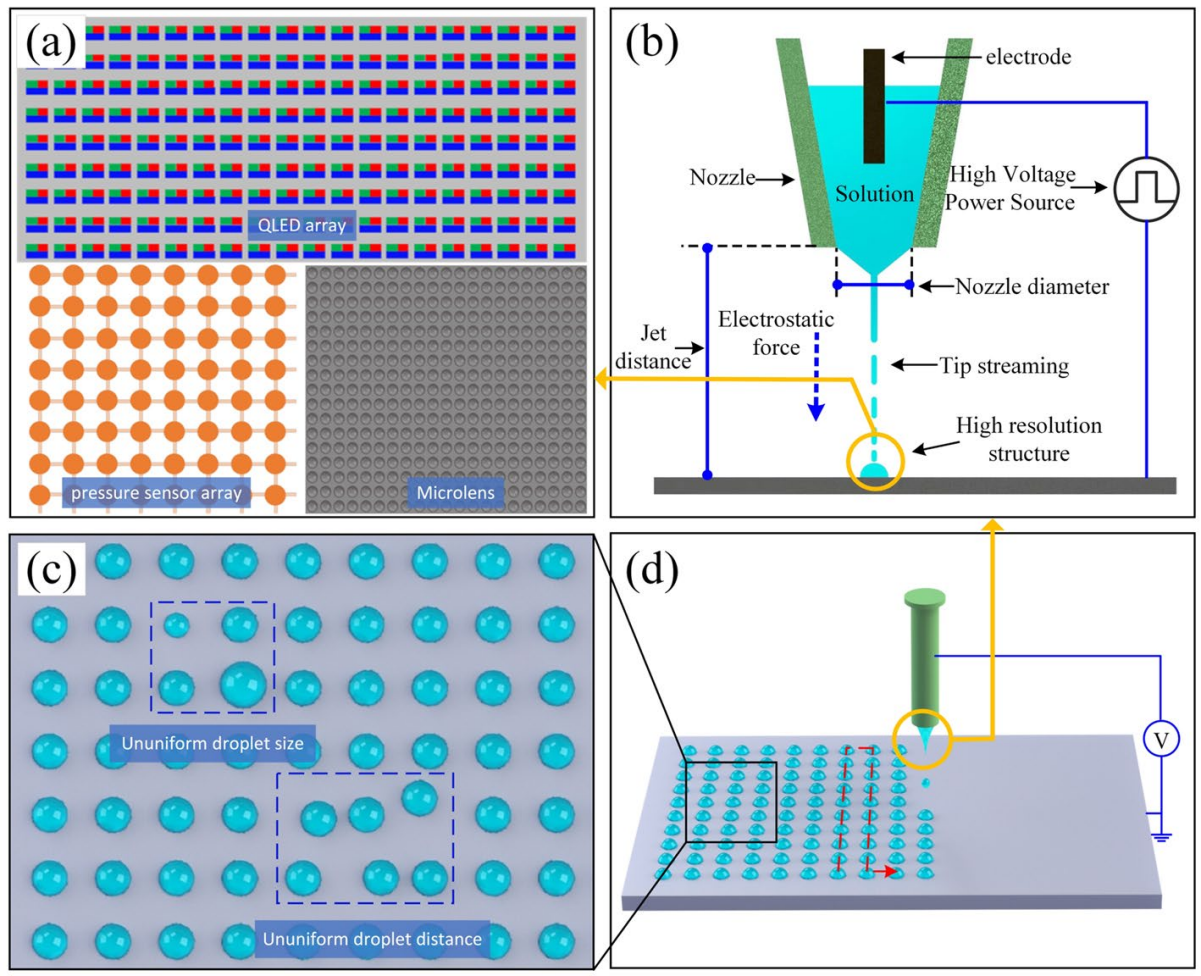
Arrayed structure components and printing defects: (**a**) high-resolution arrayed structure, (**b**) electrohydrodynamic printing principle, (**c**) printing defects, and (**d**) electrohydrodynamic printing-based high-resolution arrayed structure printing process.

The high-resolution arrays (as shown in Fig. 1a) are the basic structure of most kinds of microelectronics. They are widely used in sensors, novel displays, bionic compound eyes, microlenses, etc.^1–6^, and the demand for resolution is increasing. For example, in near-eye display applications where even more than 10,000 PPI is needed to eliminate the “screen door effect (SDE)”^7^. The current mainstream processes for preparing high-resolution arrayed structures include photolithography and e-beam lithography as well as vacuum evaporating, etc. These technologies either have low material utilization ratio or have long technological process. In recent years, inkjet printing technology has attracted wide attention in micro and nano fabrication due to its advantages of high material compatibility, outstanding pattern ability, and process efficiency^8–15^. During the printing process, the ink is accurately deposited on substrates, and the micro/nanoscale structures are formed. The inkjet printing technology has been successfully applied to high-definition screens, flexible electronics, and biomedical applications^16–21^.

Limited by small-size nozzle fabrication and the “Squeeze” injection principle^22^, it is a great challenge to prepare array structures above 500 PPI using traditional inkjet printing technology. E-jet printing technology uses an applied electric field to “pull” the droplet out of the nozzle. It is compatible with different solutions in a more extensive viscosity range (1–10,000 cPs) and can generate droplets much smaller than the nozzle diameter due to the formation of droplets at the tip of the Taylor cone, thus enabling the printing of high-resolution structures at the micro or even nanoscale^23^. By using an electro fluidic nozzle to prepare microelectronics, Koei et al. successfully printed electrodes with a minimum line width of 15 $\upmu$m on polyimide films^24^. Bong et al. performed high-resolution quantum dot ink printing and successfully printed droplets with a diameter of 3 $\upmu$m, which could be used as an active layer for QD light-emitting diodes^25^. Mishre et al. printed 1 $\upmu$m photocurable polymer and experimented with arrayed patterns, printing arrayed dots with 10 $\upmu$m diameters and 30 $\upmu$m pitch^26^. Liang et al. changed the spraying method of the nozzle^27^. They achieved the preparation of nanodroplets with an average diameter of 73 nm by “dipping” and then “spraying”, but from the results given, the uniformity of droplet diameter was poor. The difference between the smallest and largest droplet diameters under the same parameters was noticeable. Galliker et al. used a nozzle with the diameter of 1 $\upmu$m to print plasma solutions (gold nanoparticles) with a diameter of 50 nm, which were grown continuously in the third dimension to achieve a large aspect ratio of the structure. The inorganic material gold nanoparticles are easier to print at the nanoscale compared to solutions due to their physical properties^28^.

Much scholars have focused their research on single droplet and how to achieve smaller dot diameter, while more researches need to be done on how to obtain consistent arrays. The difficulties of preparing high resolution arrays are ignored. The E-jet printing technology (as shown in Fig. 1b) is promising in high resolution array printing(as shown in Fig. 1d). In the E-jet printing of high-resolution structures, the droplet generation and deposition duration are short (millisecond level), the droplet size is usually in the micro/nanoscale, and the whole process is affected by multiple physical fields. Although studies on E-jet printing have been carried out for many years, the mechanism of the jet break, droplet trajectory, and charge power during the printing process is still poorly understood. Ununiform droplet size and spacing (as shown in Fig. 1c) occur under inappropriate parameters settings, which will greatly influence the performance and lifetime of the array based microelectronics^29–33^. To improve the uniformity of the printed array, it is of vital importance to find suitable process parameters to avoid defects and achieve higher resolution.

For the requirements of high-resolution dot array structures in high-resolution microelectronics, the E-jet printing process has been widely used in preparing high-resolution arrays with its high resolution and easy patterning advantages. However, it is still challenging to achieve both high resolution and high uniformity. To improve the uniformity of droplet size and droplet pitch in the printed dot array, we carried out experiments on the self-built NEJ-E/P200 platform. We established the judging criteria of droplets volume consistency and pitch consistency. And the effects of electric voltage, supply pressure, nozzle diameter, printing height, printing speed and other parameters on the array uniformity are experimentally investigated. Finally, the dot array with resolutions of 500 PPI, 650 PPI, 1250 PPI, 1700 PPI, 3150 PPI, 5000 PPI, 7000 PPI, 12,000 PPI and 17,000 PPI with well uniformity are implemented.

## Materials and methods

### Experimental platform

**Figure 2:**
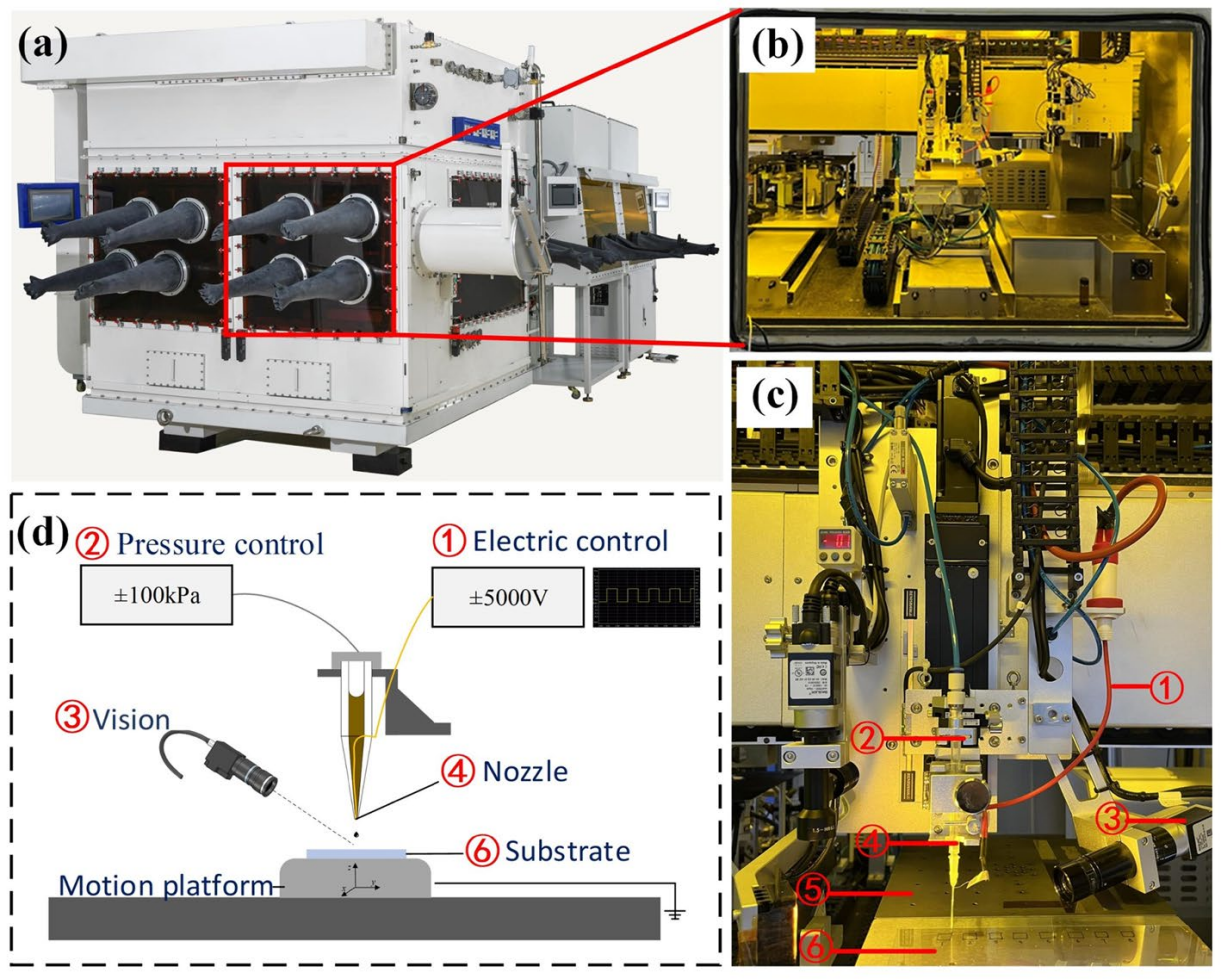
NEJ-E/P200 Experimental platform: (**a**) diagram of NEJ-E/P200 Experimental platform appearance, (**b**) partial enlargement of the equipment, (**c**) diagram of each module of the equipment, and (**d**) simplified diagram of the equipment.

The experiment was carried out on the NEJ-E/P200 platform (as shown in Fig. 2), jointly developed by Huazhong University of Science and Technology and Wuhan National Innovation Technology Optoelectronics Equipment Co., LTD. The platform has both piezoelectric inkjet printing and E-jet printing systems. Here the E-jet printing system is used, which consists of a motion control module, a voltage control module, a vision module, a pressure control module and a nozzle module. The voltage control module outputs voltage of different amplitudes, frequencies and duty ratios, and establishes an electric field between the nozzle and the substrate. The motion control module realizes various forms of motion required for printing, with a motion accuracy of 1 $\upmu$m. The vision module is responsible for real-time monitoring of the printing process. The pressure control module applies pressure to the solution of the nozzle, and adjusts the pressure range from 0–1000 mbar with an accuracy of 0.1 mbar. The nozzle module is a homemade device, mainly composed of conductive copper wire and glass nozzle, as shown in Fig. 2. The cleaned glass substrate is placed on the mobile platform of the experimental platform and fixed using an adsorption device. The position signal induced printing strategy is adopted. In order to achieve precise control of the droplet drop position, we associate the motion signal of the axis with the ejection signal. When the axis reaches the designated position, a high-voltage pulse is sent to achieve accurate ejection of the droplet at that position. The cycle of a single pulse is fixed at 1 s, the duration of high voltage is controlled by the duty ratio. A critical voltage (70% of the Bias voltage) is applied when the axes are moving from one position to another, which will not cause ejection but helps to form a stable shape of the Taylor cone.

All operations are performed in a clean room to minimize the influence of the external environment on the experimental results.

### Experimental materials

#### Solution

The solution chosen for this experiment is Epoxy resin, a non-volatile liquid. The printed pattern can exist on the substrate for a long time even at micro/nanoscale, making it easily observed. The solution parameters are shown in Table 1.

**Table 1:** Basic parameters of Epoxy resin.

Solution	Density	Viscosity	Surface tension	Conductivity
Epoxy resin	1.01 kg/m^3^	22 cp (25^∘^)	32 mN/m	< 0.001 $\upmu$/Scm

#### Nozzle

**Figure 3:**
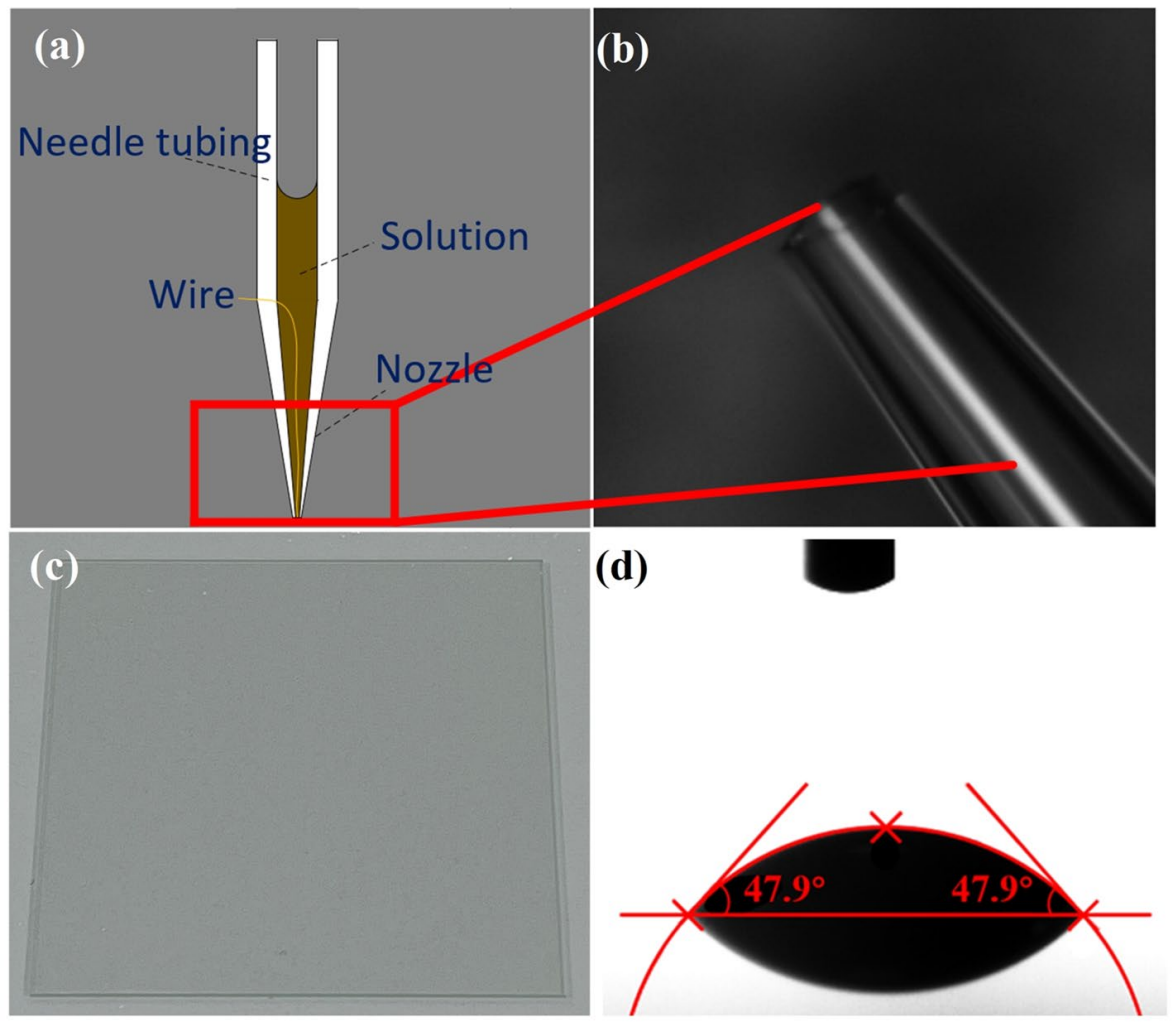
Experimental needle and substrate: (**a**) diagram of nozzle structure, (**b**) diagram of the nozzle, (**c**) diagram of the substrate, and (**d**) substrate contact angle measurement.

The nozzle structure is shown in Fig. 3a,b. Two nozzle sizes were used in the experiment with inner diameters of 2 $\upmu$m and 20 $\upmu$m respectively and were prepared using the P-1000 needle puller. The needle tubing was a medical plastic syringe with a capacity of 1 ml and the material of the wire was copper with a diameter of 100 $\upmu$m.

#### Substrate

The glass substrate (as shown in Fig. 3c) was selected as the experimental substrate with a contact angle of $47.9^{\circ }$ with the solution to be printed (as shown in Fig. 3d). The substrates were ultrasonically cleaned with ethanol to remove organic and inorganic impurities. They were then rinsed with deionized water (DI) and dried under a nitrogen atmosphere for the experiments.

### Judging criteria of the printed dot array

After the dot array is printed, the diameter of each droplet and the spacing between the droplets are detected using a White Light Interferometer. For dot arrays under the same resolution, the mean and variance of the diameters of sessile-droplets are used to assess the consistency of droplet size in the printed dot array; the average transverse and longitudinal deviations are us ed to assess the consistency of droplet spacing. The uniformity of the dot array is defined by the consistency of droplet size and droplet spacing.

**Figure 4:**
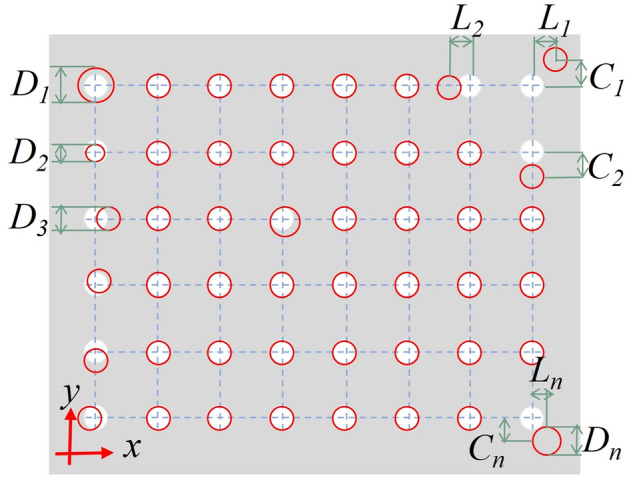
Judging criteria for arrayed patterns.

As shown in Fig. 4, $D_n$ is the diameter of each droplet,$C_n$ and $L_n$ are the transverse and longitudinal position errors. The average and variance of the droplet diameter of the printed dot array are:

1 $$\begin{aligned} \overline{D}&= \frac{(D_1+D_2+\cdots +D_n)}{n} \end{aligned}$$

2 $$\begin{aligned} \sigma ^2&=\frac{\Sigma (D-\overline{D})^2}{N} \end{aligned}$$

The average transverse and longitudinal position error are calculated as:

3 $$\begin{aligned} \overline{C}&= \frac{(C_1+C_2+\cdots +C_n)}{n} \end{aligned}$$

4 $$\begin{aligned} \overline{L}&= \frac{(L_1+L_2+\cdots +l_n)}{n} \end{aligned}$$

The more minor variance of droplet diameter and the average transverse and longitudinal deviations shows better uniformity of the printed dot array.

## Experimental results and discussion

### Influence of printing parameters to the uniformity of printed dot array

The E-jet print process is complex and influenced by a lot of problems. In this work, the effect of supply pressure, printing height, voltage and printing speed on the uniformity of the printed dot array was analysed using a 20 $\upmu$m nozzle. The default experimental parameters are listed in Table 2. Hereafter, the parameters listed in Table 2 are used in all cases unless otherwise specified.

**Table 2:** The default experimental parameters.

Parameter	Data
Nozzle diameter	20 $\upmu$m
Supply pressure	10 kPa
Printing height	200 $\upmu$m
Bias voltage	400 V
Voltage amplitude	100 V
Duty	30%
Travel speed	10 $\upmu$m/s

### Influence of supply pressure

**Figure 5:**
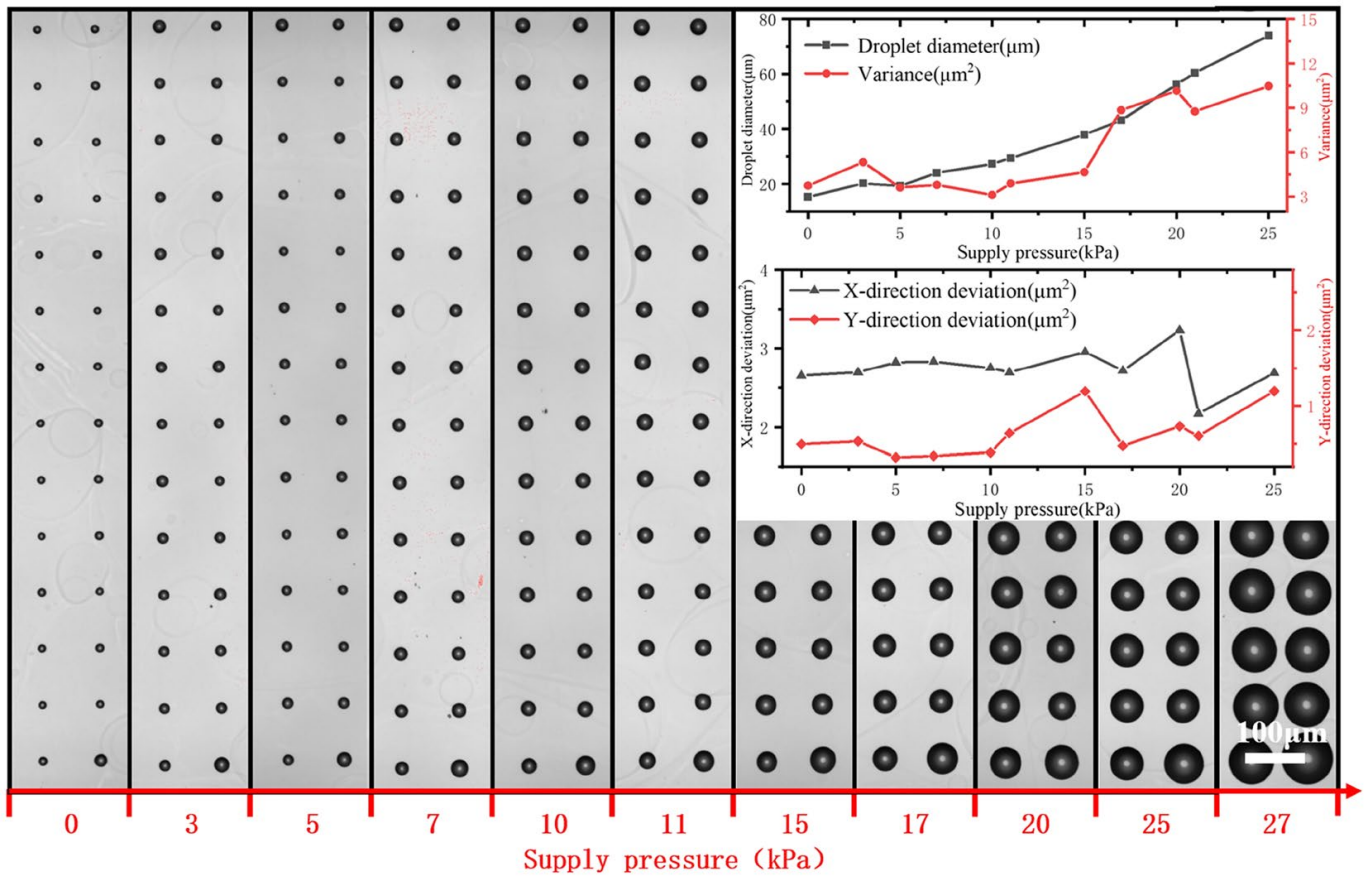
Effect of supply pressure on the uniformity of printed array.

The printed dot arrays and the uniformities under different supply pressures are shown in Fig. 5. With the increase of supply pressure, the droplet diameter steadily increases and the two become approximately linearly correlated. The droplet diameter variance, transverse and longitudinal deviation values are smaller at lower pressure, showing better uniformity. When the pressure is greater than 15 kPa, the Y-directional (longitudinal direction) deviation of the arrayed droplets increases first, and then the X-directional deviation (transverse) and the droplet variance both start to increase.

### Influence of printing height

**Figure 6:**
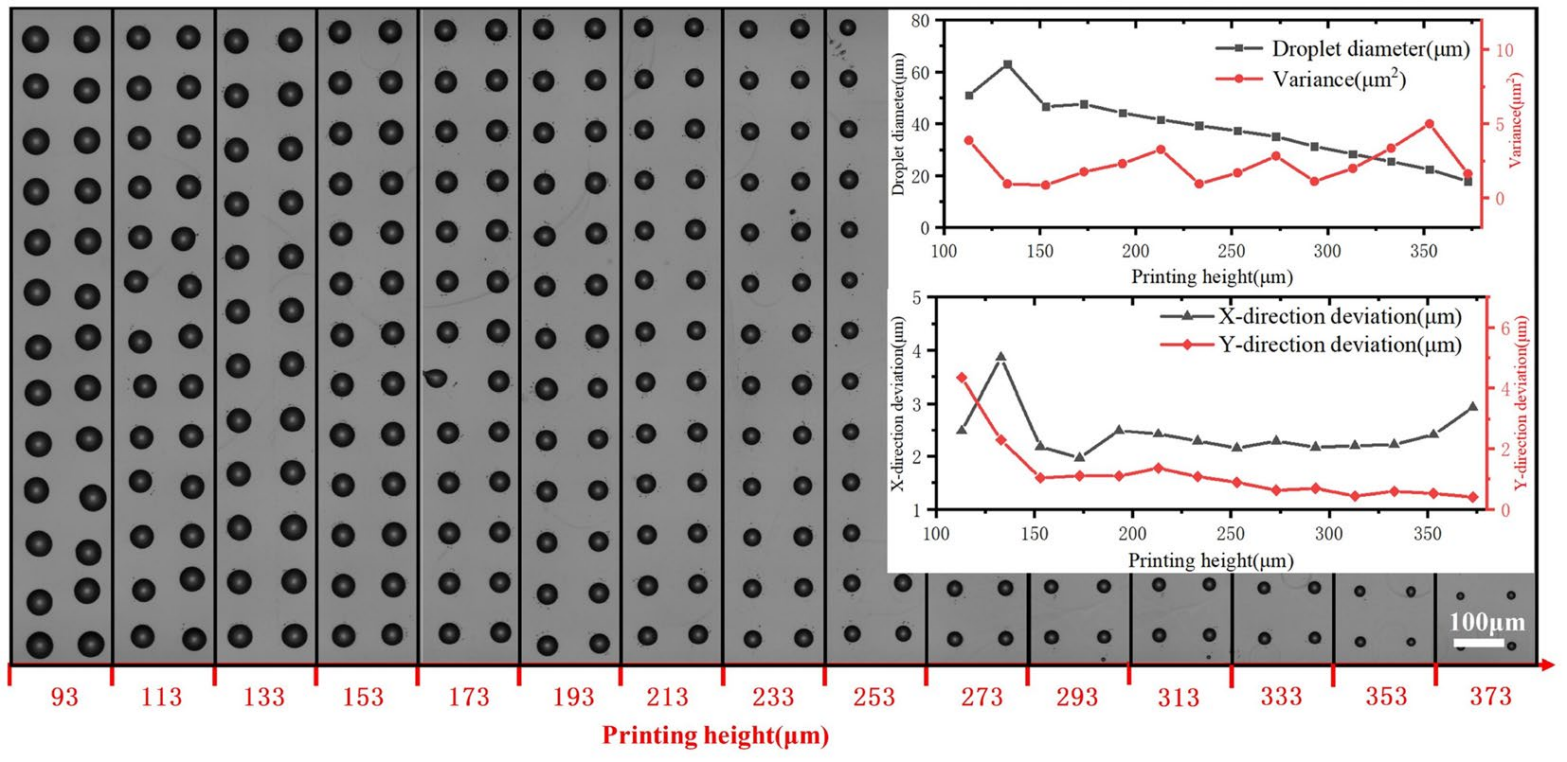
Effect of printing height on the printed dot array.

The printed dot arrays and the uniformities under different printing heights are shown in Fig. 6. Within a reasonable range of printing height, the droplet diameter gradually decreases with increasing printing height, while the variances of the droplet diameter only fluctuate within 5 $\upmu$m^2^. The electric field is formed between the electrode and the platform, as the electrode is fixed in the nozzle, increasing the printing height will decrease the intensity of the electric field and the surface charge repulsion force in the Taylor cone, thus the volume of the printed droplet becomes smaller. The x direction and y direction deviation are generally decrease with the increase of the printing height. In the case of low printing height, the electric field strength is relatively large, the volume of the droplet and the charge carried by the droplet are larger, and the crosstalk effect between the droplets is intensified in the case of larger electric field strength and larger droplet charge, which in turn leads to the aggravation of the drop positioning error. The differences between the x direction and y direction is mainly cause by the micro vibration of the axes in the stop and go process in the printing direction (x direction). Increase printing height will enlarge the x direction deviation due to the vibration of the axes. An appropriate print height is required to improve the uniformity of printed dot array.

At lower printing heights, the X-directional and Y-directional deviation are more significant. When the print height is higher than 150 $\upmu$m, the deviation starts to decrease, but when the print height is higher than 350 $\upmu$m, the X-directional deviation starts to increase again, and the X-directional stability starts to decrease.

Different printing heights only affect the diameter of the printed droplets and have a minor influence on the uniformity of the droplet diameter. When the printing height is within the range of 150–350 $\upmu$m, it almost does not affect the X-direction and Y-direction deviation, but when it exceeds the range, the uniformity decreases.

### Influence of voltage

Voltage plays the most critical role in the diameter and stability of the jet, and the experiments are conducted using a pulsed DC voltage, where the basic parameters of the pulsed DC voltage are bias voltage, voltage amplitude, duty ratio, and frequency.

**Figure 7:**
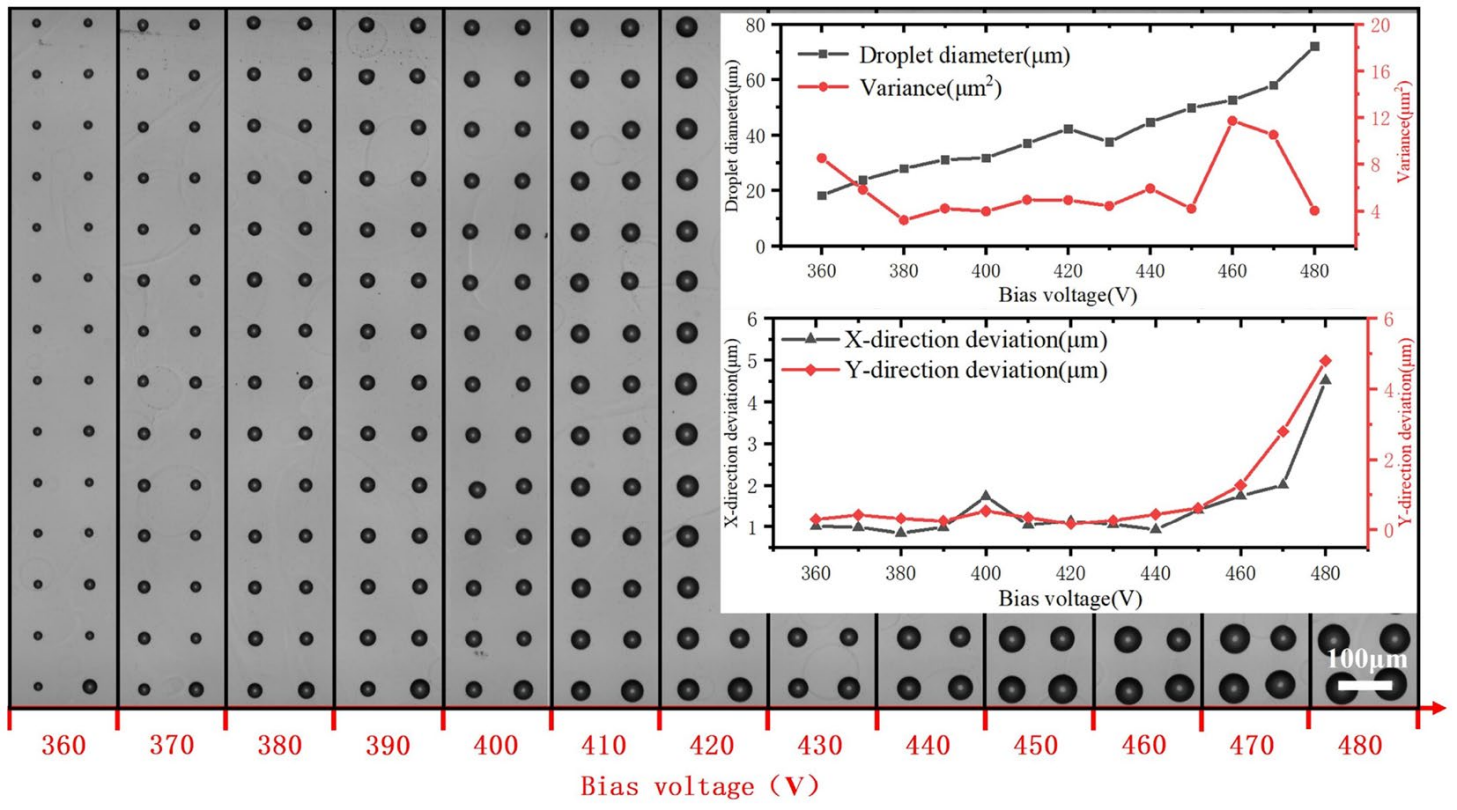
Effect of bias voltage on the printed dot array.

The printed dot arrays and the uniformities under different bias voltages are shown in Fig. 7. When the bias voltage is low, the droplet is challenging to be ejected, and the droplet diameter increases gradually with the bias voltage in a suitable range. As the position signal induced printing strategy is adopted, the increase in both bias voltage and voltage amplitude will lead to an increase in the ejection voltage. In the droplet ejection process, as the ejection voltage increases, the greater voltage produces more charges on the surface of the Taylor cone, and the greater electric field force causes larger deformation of the Taylor cone, so the volume of the droplet is larger. In the deposition process, larger ejection voltage will cause larger crosstalk effect thus increases the droplet positioning error. The variance of droplet diameter is smaller between 380 and 450 V, which shows a better uniformity of the dot array. X-direction and Y-direction deviations are minor at lower bias voltages, and the uniformity of the arrays severely decreases when the bias voltage is over 450 V.

**Figure 8:**
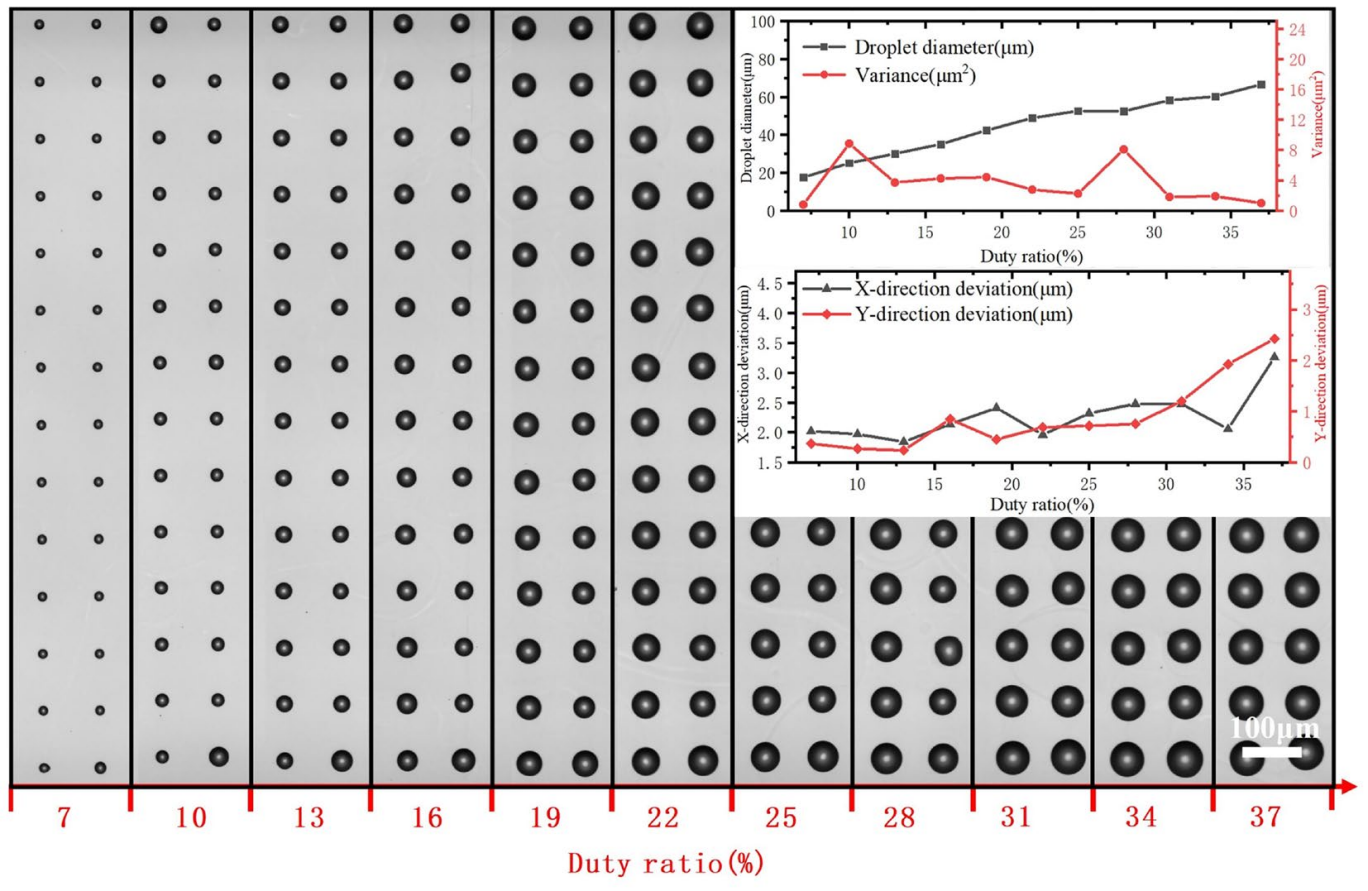
Effect of duty ratio on the printed dot array.

The printed dot arrays and the uniformities under different Duty ratios are shown in Fig. 8. With the increase in duty ratio, the droplet diameter gradually increases, while the variations of the droplet diameter tend to decrease. Increasing duty ratio increase the duration of the deformation of Taylor cone, which will ultimately lead to an increase in the deformation of Taylor cone and therefore an increase in droplet volume. For better consistency of droplet diameter, a slightly larger duty ratio is recommended. X-direction and Y-direction deviation are increased with the increase of the duty ratio. To acquire both well consistency of droplet diameter and spacing, a suitable duty ratio is needed.

Voltage plays a vital role in electrohydrodynamic printing, and is the core element to achieve the injection as well as uniformity or not. The matching of bias voltage and voltage amplitude is essential. After repeated experiments have proven that the peak voltage in a cycle to ensure that the nozzle spraying, non-peak voltage period (occupancy), to ensure that the nozzle has been in the state of critical injection is also essential, to ensure that the next peak voltage, to achieve immediate injection, thereby ensuring the consistency of the overall volume of sprayed droplets. For different solutions and needle sizes, multiple attempts to find the critical injection voltage is most important.

### Influence of travel speed

**Figure 9:**
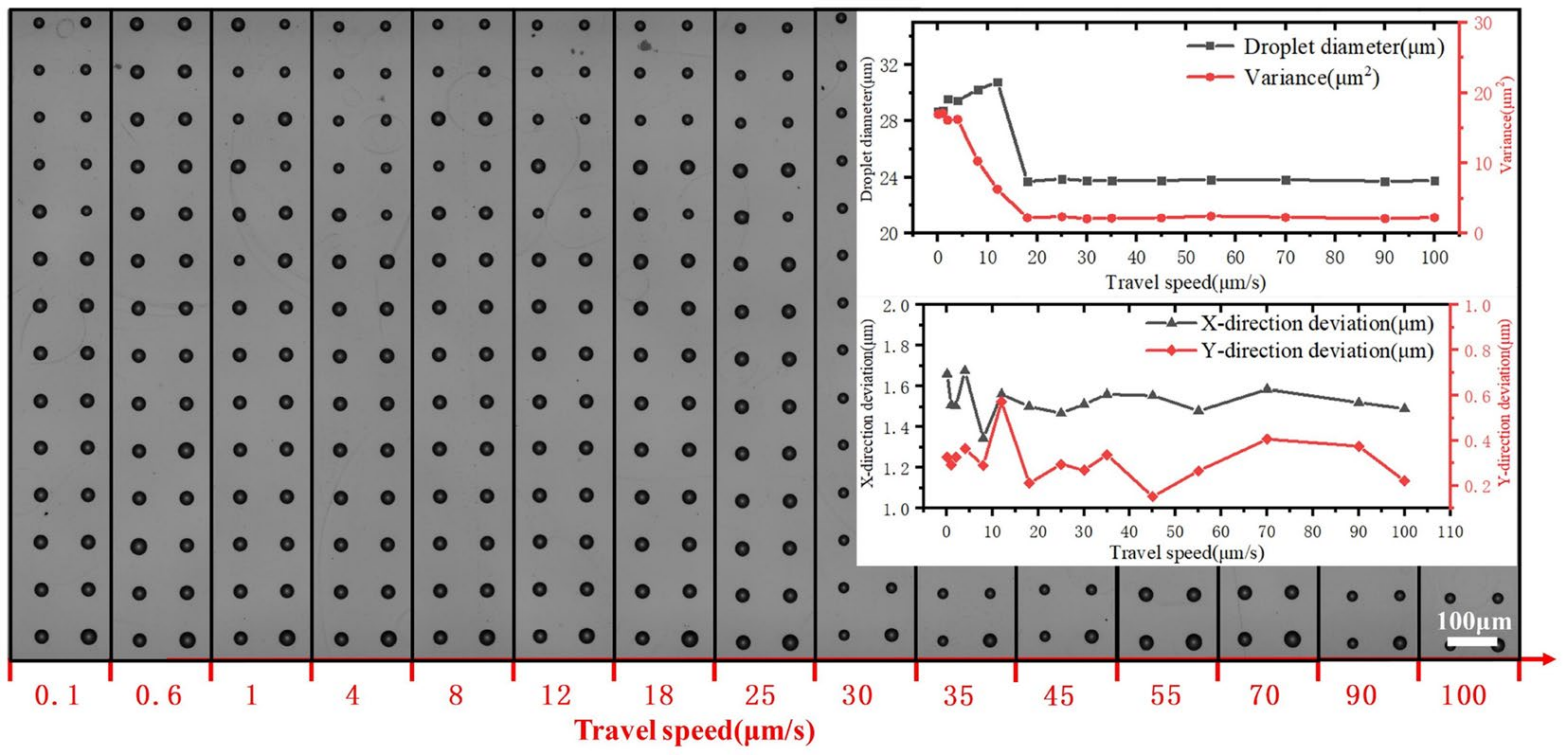
Effect of travel speed on printed dot array.

The printed dot arrays and the uniformities under different travel speeds are shown in Fig. 9. The moving speed of the experimental platform influences the efficiency of dot array preparation. Experiments were conducted with a moving speed of 0–100 $\upmu$m/s. As can be seen from the graph, at lower moving speeds, the print droplet diameter is higher, the droplet diameter variance is also higher, and the droplet printed is not uniform. When the printing speed exceeds 20 $\upmu$m/s, the consistency printed droplet is better. X-direction and Y-direction deviation with the increase of printing speed are within 0.8 $\upmu$m, the printing speed has less impact on the overall stability.

### summary of the influences

In summary, as the needle pressure increases, the droplet diameter gradually increases, and the consistency of the arrayed pattern is lower; within the suitable nozzle height range, as the nozzle height decreases, the droplet diameter gradually decreases, and the uniformity of the printed dot array pattern is better; the influence of the electric field voltage on the results is more evident and complex, within the suitable voltage range, as the bias voltage, voltage amplitude and duty ratio increase, the droplet diameter gradually increases, while the stability of the arrayed pattern becomes worse; with the increase of moving speed, the droplet diameter gradually decreases, and the stability of the arrayed pattern becomes better. The overall trend is shown in Table 3, and the graph guides high-resolution dot array printing.

**Table 3:** Effect of process parameters on arrayed printing.

Parameters	Pressure	Height	Bias voltage	Voltage amplitude	Duty ratio	Travel speed
Diameter	$\nearrow$	$\searrow$	$\nearrow$	$\nearrow$	$\nearrow$	$\searrow$
Variance	$\nearrow$	−	$\nearrow$	$\nearrow$	−	$\searrow$
X-deviation	$\nearrow$	$\searrow$	$\nearrow$	$\nearrow$	$\nearrow$	−
Y-deviation	$\nearrow$	$\searrow$	$\nearrow$	$\nearrow$	$\nearrow$	−

For the main influencing factors of the printing state, such as supply pressure, bias voltage, and printing height, we constructed a database of the effects of supply pressure, bias voltage, and printing height on the changes of the deposited droplet diameter and the deviation in the x-direction and y-direction based on the experimental data, and used the MLP to fit the training data to get the data model under the multi-parameter coupling. Where the input layer of the MLP training model is 3, three hidden layers are used, and the number of neurons in each layer is 50, 50, and 25, respectively, and the R-squared score between the prediction result and the real target variable is 0.9105, which has a good fitting result.

**Figure 10:**
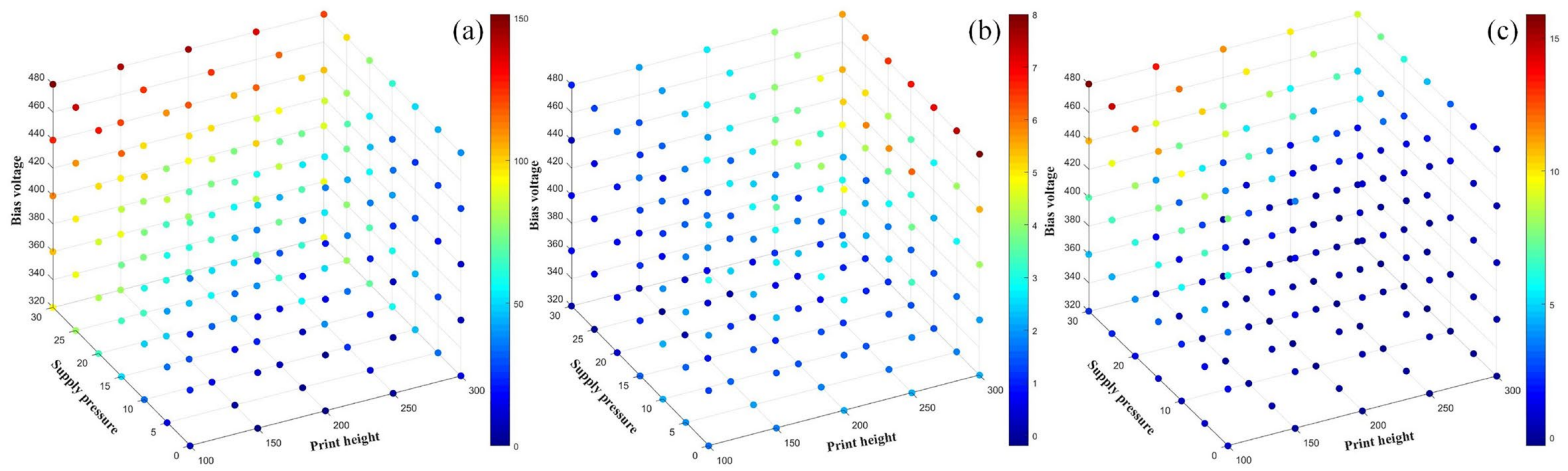
Process phase diagrams for each parameter: (**a**) variation of droplet volume with process parameters, (**b**) variation of droplet x-direction deviation with process parameters, (**c**) variation of droplet y-direction deviation with process parameters.

The variation of droplet diameter, droplet x-direction deviation and y-direction deviation with process parameters (supply pressure, bias voltage, print height) can be seen in Fig. 10, which can guide the selection of process parameters in the printing process. For high resolution dot array printing, smaller dot size and droplet positioning error are needed. As shown in the process window, the printing parameters with smaller supply pressure, smaller bias voltage and relatively lager printing height are recommended. The construction of a coupled model about supply pressure, bias voltage, and print height helps to determine the optimal process window, which is a good guide for the actual printing process.

### Printed dot arrays with different resolutions

**Figure 11:**
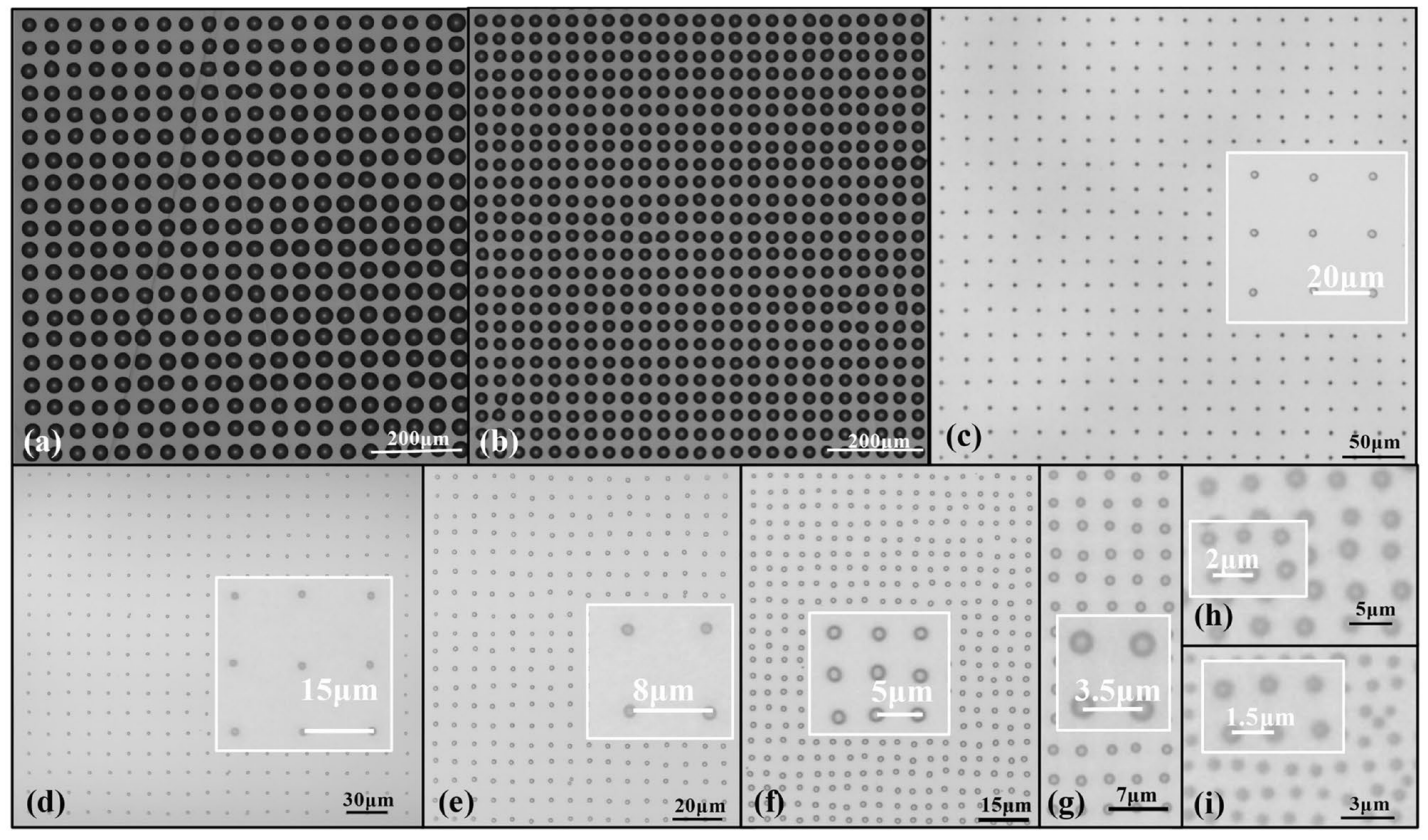
Printing results with different resolutions: (**a**) 500 PPI, (**b**) 650 PPI, (**c**) 1250 PPI, (**d**) 1700 PPI, (**e**) 3150 PPI, (**f**) 5000 PPI, (**g**) 7000 PPI, (**h**) 12,000 PPI, and (**i**) 17,000 PPI.

The apparent impact of each parameter on the printing effect is more indicative of the controllability of E-jet printing technology. In addition to exploring the impact of each parameter on droplets and arraying, it is also worth discussing the issue of adjusting process parameters to achieve smaller droplet volumes and higher resolutions. This paper explores the limits of printing resolution on a NEJ-E/P200 Experimental platform. The results shown in Fig. 11a,b are printed with nozzle diameters of 20 $\upmu$m, the rest results are printed with nozzle diameters of 2 $\upmu$m. The experimental parameters of the printed dot arrays are listed in Table 4.

**Table 4:** The experimental parameters of the printed dot arrays.

PPI	Nozzle diameter ($\upmu$m)	Supply pressure (kPa)	Printing height ($\upmu$m)	Bisa voltage (V)	Voltage amplitude (V)	Duty ratio (%)	Travel speed ($\upmu$m)
500	20	0.7	200	300	200	30	50
650	20	0.7	200	300	200	30	20
1250	2	5	150	300	100	50	5
1250	2	3	150	300	100	50	5
1250	2	3	100	300	50	35	2
1250	2	3	90	300	50	35	2
1250	2	3	80	300	50	35	2
1250	2	2	60	280	50	20	1
17,000	2	1	60	250	50	20	1

**Figure 12:**
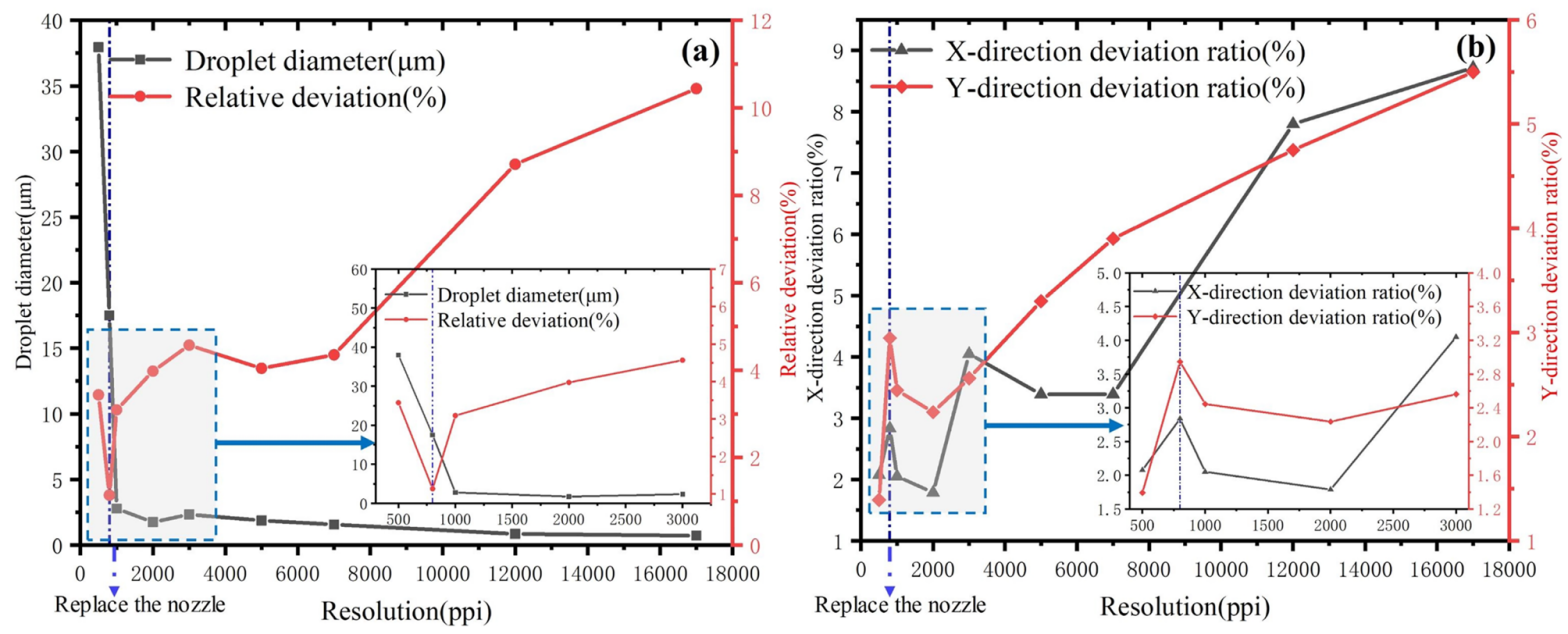
Uniformities of printed dot array at different resolutions: (**a**) droplet diameter and deviation with different resolutions, and (**b**) X and Y-direction deviation rates with different resolutions.

The printed dot arrays and their uniformities are shown in Figs. 11 and 12. As shown in Fig. 12a, the droplet diameter is mainly influenced by the nozzle and process parameters; at a lower resolution, the droplet variance is slight, and the print pattern’s droplet volume consistency is better now. When the resolution exceeds 5000 PPI, the volume variance starts to increase gradually. When it exceeds 12,000 PPI, the volume consistency is getting worse, but the relative deviation is within 10% overall. The consistency of the droplet volume is mainly limited by the accuracy of the supply pressure, as the volume of dots in high resolution array are only several femtoliters, even a small disturbance in supply pressure will influence the printed dot size. As shown in Fig. 12b, the X- direction and Y-direction deviation rates are the ratio of X- direction and Y-directional deviation to its spacing, which can indicate the uniformity of dot spacing at different resolutions. At lower resolutions, the stability of the position is still guaranteed, and as the resolution increases, different needles appear less stable. When the resolution exceeds 15,000 PPI, the X-direction deviation has been more than 10%, the Y-direction is still within 6%.

On the one hand, the printing of a dot array is limited by the nozzle diameter and process parameters; on the other hand, the motion accuracy of the equipment is also important. Moreover, with increase of the resolution, the printing stability is also influenced by the cross-talk effect, which need to be further considered. The current NEJ-E/P200 platform has a moving accuracy of 1 $\upmu$m, and the 17,000 PPI (spacing of 1.5 $\upmu$m) dot array is currently prepared, has reached one of the highest resolutions of dot arrays by printing reported at present (as shown in Table 5). In the premise of improving the accuracy of the equipment, it is expected to achieve more than 20,000 PPI pattern printing.

**Table 5:** Resolution of printed dot array.

Printing method	Resolution (PPI)	References
E-jet printing	17,000	This work
E-jet printing	12,000	^34^
E-jet printing	5000	^35^
Transfer printing	2460	^2^
E-jet printing	2100	^3^
Piezoelectric inkjet	1000	^36^
Piezoelectric inkjet	846	^37^
Piezoelectric inkjet	500	^38^
Piezoelectric inkjet	380	^39^
Piezoelectric inkjet	228	^40^
Piezoelectric inkjet	120	^41^
Piezoelectric inkjet	113	^42^
Piezoelectric inkjet	85	^43^

## Conclusion

Electrohydrodynamic printing process is widely used in the preparation of high-resolution array structures with its advantages of high resolution and easy patterning. However, it is still challenging to achieve high resolution while ensuring good uniformity. In this paper, the influence of process parameters on the uniformity of printed arrays is studied, and high-resolution arrays are implemented. The main conclusions are as follows:

- The influences of process parameters on the uniformity of arrays are studied. We investigated the influence of process parameters such as electric voltage, supply pressure, printing height, and travel speed on the consistency of droplet diameter and droplet spacing with the help of NEJ-E/P200 experimental platform, and found out the critical role of electric field voltage in maintaining the stability of printed dot array.
- The high-resolution dot arrays are printed. Based on the experimental results, the dot arrays with resolutions of 500 PPI, 650 PPI, 1250 PPI, 1700 PPI, 3150 PPI, 5000 PPI, 7000 PPI, 12,000 PPI and 17,000 PPI (spacing of 1.5 $\upmu$m with a diameter of 700 nm) are successfully printed by adjusting each process parameter. For arrays below 10,000 PPI, the droplet diameter and pitch deviations are within 5%, and for arrays above 10,000 PPI, the consistency decreases but remains within 10% overall.

This paper is the first to realize the dot array of more than 15,000 PPI based on E-jet printing. The current 17,000 PPI dot array has already close to the limit of the experimental platform’s motion accuracy. The dot array preparation of more than 20,000 PPI is printable by improving the motion accuracy of the platform. The conclusion of the effect of different parameters on array consistency explored in this paper is of great significance for E-jet printing preparation of high-resolution arrayed structures.

## Data Availability

All data generated or analysed during this study are included in this published article.
